# Longitudinal examination of changing fertility intentions and behaviors over a four-year period in urban Senegal

**DOI:** 10.1186/s12978-020-0893-4

**Published:** 2020-03-17

**Authors:** Ilene S. Speizer, Veronica Escamilla, Peter M. Lance, David K. Guilkey

**Affiliations:** 1 Department of Maternal and Child Health, Gillings School of Global Public Health, University of North Carolina at Chapel Hill, Chapel Hill, NC USA; 2 Carolina Population Center, University of North Carolina at Chapel Hill, 123 W. Franklin St, Chapel Hill, NC 27516 USA; 3 Department of Economics, Carolina Population Center, University of North Carolina at Chapel Hill, Chapel Hill, NC USA

**Keywords:** Fertility, Family planning, Senegal, Pregnancy intentions, Longitudinal

## Abstract

**Background:**

Fertility intentions and contraceptive use are often used to demonstrate gaps in programs and policies to meet the contraceptive needs of women and couples. Prior work demonstrated that fertility intentions are fluid and change over a woman’s (or couple’s) life course with changing marital status, childbearing, and education/employment opportunities. This study uses longitudinal data to better examine the fluidity of women’s fertility intentions and disentangle the complex interrelationships between fertility and contraceptive use.

**Methods:**

Using survey data from three time points and three urban sites in Senegal, this study examines how women’s fertility intentions and contraceptive use in an earlier period affect pregnancy experience and the intentionality of experienced pregnancies among a sample of 1050 women who were in union at all three time points. We apply correlated random effect longitudinal regression methods to predict a subsequent birth by fertility intentions and modern contraceptive use at an earlier period addressing endogeneity concerns of earlier analyses that only include two time periods.

**Results:**

Descriptive results demonstrate some change in fertility desires over time such that 6–8% of women who reported their pregnancy as intended (i.e., wanted to get pregnant at time of pregnancy) reported earlier that they did not want any(more) children. Multivariate analyses demonstrate that women who want to delay or avoid a pregnancy and are using modern contraception are the least likely to get pregnant. Among women who became pregnant, the only factor differentiating whether the pregnancy is reported as intended or unintended (mistimed or unwanted) was prior fertility intention. Women who wanted to delay a pregnancy previously were more likely to report the pregnancy as unintended compared to women who wanted to get pregnant soon.

**Conclusions:**

These results suggest some post-hoc rationalization among women who are getting pregnant. Women who say they do not want to get pregnant may be choosing not to use a contraceptive method in this urban Senegal context of high fertility. Programs seeking to reach these women need to consider their complex situations including their fertility intentions, family planning use, and the community norms within which they are reporting these intentions and behaviors.

## Plain English summary

Women’s fertility intentions change over their life course with changes in relationship status, childbearing experience, and education or employment opportunities. This study uses data from 1050 women surveyed at three time points to examine how women’s fertility intentions change over time and to disentangle the complex interrelationships between fertility desires and contraceptive use. Women included in the study lived in three cities in Senegal and were married at the time of each survey round. Study results show that women change their fertility desires over time. About 7 % of women who said that they did not want to get pregnant in the future report an experienced pregnancy in the follow-up period as wanted at the time of the pregnancy. Multivariate analyses show that fertility desires (i.e., the desire to delay or avoid a pregnancy) and contraceptive use are strong predictors of pregnancy experience in the follow-up period. Those women who acted on their fertility desires (e.g., wanted to delay or avoid a pregnancy) by using contraception were the least likely to experience a pregnancy over time. In the urban Senegal context with continued high fertility, women who experience a pregnancy, whether desired or not, may report the pregnancy retrospectively as intended. Programs need to help women who desire to delay or avoid a pregnancy meet their intended fertility desires through equitable access to effective contraceptive methods.

## Background

Unintended pregnancy remains a global challenge. In 2012, an estimated 85 million unintended pregnancies occurred worldwide [1]. The highest regional rate of unintended pregnancy among women ages 15–44 years occurred in Africa (80 unintended pregnancies/1000 women) [1]. Within Africa, the highest sub-regional rate was 108 unintended pregnancies per 1000 women in both Eastern and Middle Africa [1]. Recent modeling of unintended pregnancy rates globally demonstrates declines in unintended pregnancy rates over time; however, the amount of change was smaller in developing as compared to developed countries [2]. Unintended pregnancy is a global concern since women who experience an unintended pregnancy may resort to unsafe abortion and unintended pregnancy may be a cause or consequence of socio-economic inequities [2].

Estimates from 2017 indicate that 214 million women in the developing world have an unmet need for modern contraception, that is they are sexually active, fecund, want to delay or limit childbearing, and are not using modern contraception [3]. Unmet need is a particular problem in sub-Saharan Africa where the proportion of women with unmet need is 21% [3]. Common reasons for unmet need in the context of high unmet need and low contraceptive use, as in the case of Senegal, include: (a) concerns about side effects/health risks of family planning; (b) opposition of the woman to contraceptive use; (c) perceived opposition to contraception by another person (e.g., her partner or a religious leader); (d) postpartum amenorrhea/breastfeeding; and (d) infrequent or no sex [4].

In Senegal, the site of this study, unmet need has declined over the study period from 29.4% of women in union in the 2010–11 Demographic and Health Survey (DHS) [5] to 22.5% in the 2015 DHS [6]. This may partly be due to investments in family planning. Family planning programming is one of the most effective ways of reducing unintended pregnancies and improving the health and well-being of women and households [3].

Earlier studies demonstrate that women may be ambivalent about a pregnancy and this might affect their use (or non-use) of contraception [7, 8]. Women who are ambivalent about pregnancy may be considered to have an unmet need for contraception (i.e., may be classified as not wanting to get pregnant and not using contraception) but may make a conscious choice not to use contraception [9, 10]. Prior research suggests that fertility intentions fall on a continuum and measuring intent as a binary outcome may be inaccurate [11]. Speizer and Lance [10] found that “wanting” vs “not wanting” a pregnancy is a false dichotomy, with many women likely falling somewhere in between. Fertility intentions are fluid, changing over time with pregnancy experiences, relationship changes, and other life events (e.g., job or school).

In Senegal, our study setting, in 2010–2011 at the time of the baseline data collection, 24.5% of pregnancies that occurred in the preceding 5 years were reported to be unintended (i.e., 20.4% mistimed and 4.1% unwanted) [5]. By 2015, at the end of the study period, the percentage of recent births that were unintended was 20% with 17.0% mistimed and 3.4% unwanted [6]. Further, in urban areas in 2010–2011, women were having on average 3.9 births per woman whereas the desired family size was 2.6 births per woman [5]. By 2015, urban women’s level of fertility had declined to 3.5 births per woman on average and the desired fertility rate was 3.1 births [6]. In 2010–2011, 20 % of urban women were using a modern method of contraception and 30% of them had an unmet need for contraception [5]; these values changed by 2015 to 30% using a modern method and 23% having an unmet need [6].

Using unique and rich longitudinal data, the aim of this study is to examine the relationships between pregnancy experience, contraceptive use, and fertility intentions. It also permits examination of how these relationships depend on other factors such as parity, marital status, and employment. Aside from the obvious advantage of allowing us to observe directly the fluidity of fertility intentions, a key advantage of such a rich longitudinal sample is that it accommodates panel data estimation methods that allow us to control for selectivity and disentangle the complex interrelationships between these variables. This study allows us to understand far better the dynamics behind fertility and contraceptive use and make recommendations for strengthening programs that aim to meet the contraceptive needs of women in need.

## Materials and methods

### Data

Our study uses data from the Measurement, Learning & Evaluation (MLE) project, the evaluation arm of the Urban Reproductive Health Initiative (URHI) funded by the Bill & Melinda Gates Foundation. More information on the MLE evaluation design and results from Senegal can be found elsewhere [12]. Longitudinal data were collected from a representative sample of women of reproductive age (15–49 years) in six urban sites of Senegal using a two-stage sampling design. (The sites were selected by the implementing partners (a consortium led by IntraHealth International) and the donor prior to baseline data collection.) For the 2011 baseline sample selection, in the first stage we used census districts as primary sampling units (PSUs) and randomly selected PSUs from each study city. We then randomly selected households from the selected PSUs (see [12] for detailed sampling description). All women of reproductive age were eligible to participate following written informed consent. Surveys were implemented at baseline (2011), midterm (2013), and endline (2015). At baseline, 9614 women completed interviews (an 89% response rate). These women came from the following six urban sites: Dakar, Mbao, Guédiawaye, Pikine, Mbour, and Kaolack. At midterm, data were collected from a sub-sample of urban sites (Mbao, Guédiawaye, and Pikine). At endline, all women who were not visitors in the household at baseline were eligible for re-interview in all six baseline urban sites. At endline, 73.5% of eligible women were successfully re-interviewed. This analysis focuses on women from Mbao, Guédiawaye, and Pikine who were interviewed in all three rounds – baseline, midterm, and endline. There was a total of 2289 such women. We focus on women in union, removing women who were not in union at all of the survey rounds (*n* = 1083). An additional 110 women were removed from the sample, of which the majority (*n* = 95) reported being infecund, and the remainder (*n* = 15) reported being sterilized. An additional 46 women were excluded because their reported fertility intention was undecided or missing. The sample used for this analysis is a balanced panel sample that includes 1050 women in union from the three time points. As can be seen in Table 1, the comparison of the balanced panel to the full sample of women in union from the three study sites in each round demonstrates that the two samples are similar at each time period and thus the analysis sample can be considered to represent women in union in the three urban sites of Senegal.

**Table 1:** Weighted percentages by demographic characteristics, contraceptive use, and subsequent birth/pregnancy for women in union at baseline (2011), midterm (2013) and endline (2015) in three study cities in Senegal

	Full baseline sample^a^	Baseline longitudinal analysis sample^a^	Full midterm sample^b^	Midterm longitudinal analysis sample^b^	Full endline sample^c^	Endline longitudinal analysis sample^c^
Sample size (N)	1901	1050	1609	1050	1517	1050
Baseline city of residence^d^						
Guediawaye	22.25	23.46	22.83	23.61	22.07	21.87
Pikine	26.14	28.84	25.81	28.37	25.49	26.59
Mbao	51.61	47.70	49.87	46.95	48.35	48.90
Age group (%)						
15–19	6.31	4.37	3.00	2.26	0.85	0.22
20–24	15.97	16.56	14.18	10.90	11.33	5.03
25–29	19.75	20.71	19.12	20.71	23.37	22.14
30–34	19.58	22.04	18.59	21.36	18.98	20.78
35–39	18.00	17.47	19.52	21.18	19.51	21.91
40–44	13.46	13.76	14.40	13.69	12.84	14.82
45+	6.93	5.07	9.59	9.90	9.73	15.10
Education (%)						
None	44.66	42.11	41.65	43.15	42.49	43.91
Primary	38.02	39.68	38.10	38.96	37.45	39.33
Secondary	15.52	16.74	16.60	14.79	16.39	14.12
≥ Higher	1.80	1.47	3.66	3.101	3.61	2.64
Wealth index (%)^e^						
Poorest	18.59	14.62	15.46	15.08	20.71	20.00
Poor	21.11	21.78	23.34	21.37	21.35	21.02
Middle	25.14	26.64	26.79	26.92	20.12	20.93
Rich	18.96	19.35	18.54	19.24	24.11	24.72
Richest	16.21	17.61	15.88	17.39	13.71	13.33
Parity (%)						
0	12.69	10.84	9.78	4.49	9.01	3.16
1	17.78	17.13	16.79	13.69	15.39	8.76
2+	69.53	72.03	73.43	81.82	75.61	88.09
Modern contraceptive use (%)^f^						
Non-user	73.14	67.90	65.40	59.39	60.26	57.06
Long-acting method	2.71	2.75	7.96	9.58	14.44	15.40
Short-acting method	21.07	25.54	23.65	27.74	22.51	23.97
Other modern	0.44	0.32	0.39	0.46	0.02	0.03
Traditional method	2.63	3.50	2.59	2.82	2.77	3.55
Intentions for future pregnancy (%)						
Want Now/≤2 years	46.43	41.97	41.61	37.21	45.29	44.02
Want to delay >2 years	32.71	37.18	33.54	36.09	28.55	25.52
No more	20.86	20.85	24.85	26.71	26.15	30.45
Experienced birth/pregnancy in period between baseline-midterm or midterm-endline(%)	N/A	N/A	51.23	53.19	45.90	42.61

Note: Women who become sterilized or infecund are excluded from analysis.

^a^Uses baseline weights

^b^Uses midterm weights

^c^Uses endline weights

^d^Small percentage of respondents living and surveyed in different cities at follow-up

^e^Wealth index calculated as quintiles using principal components analysis based on assets reported in the household survey

^f^Long-acting methods include implants and IUD; spacing methods include daily pill, emergency pill, injectables, and condoms; other modern includes LAM and spermicide/mousse/gel

For each woman, we examine her fertility desires and contraceptive behaviors, at the earlier time period and relate this to her fertility experiences by the later time period. We use two transition horizons: baseline-midterm and midterm-endline. At each round of data collection, questions were asked about pregnancy and birth experiences in the last 2 years. All women who had a birth or were currently pregnant at the time of the survey (i.e., at midterm or endline), were asked about the intentionality of the birth (or pregnancy).

### Outcome variables

The primary outcome for this analysis is a subsequent birth/current pregnancy in the survey interval baseline-midterm or midterm-endline. All women who reported a birth in the two-years between surveys as well as the women who were currently pregnant at the time of the survey (midterm or endline) are classified as experiencing a subsequent pregnancy; all others are classified with no pregnancy in the interval.

Our second outcome of interest captures pregnancy experience and intentionality of an experienced pregnancy/birth. Women are classified into three categories. The first category did not have a pregnancy in the two-year period. The second category is for women who became pregnant and reported wanting the child/pregnancy when they became pregnant (intended pregnancy). The third category is for women who became pregnant but reported wanting to delay the pregnancy or wanting no(more) children (unintended pregnancy).

The key predictor variables for these analyses are the fertility desires and contraceptive behaviors in the earlier period. At baseline and midterm, women were asked if they wanted to get pregnant in the future and if so, when they would like this future pregnancy. All women who reported that they wanted to get pregnant soon or in the next 2 years were coded as wanting to get pregnant now (category 1). These women are not considered to have an imminent need for contraception given that it might take a few months to get pregnant and the pregnancy would last 9 months. Women who reported wanting to get pregnant but did not know when were also coded as wanting to get pregnant now (category 1) since they are not conscientiously trying to avoid a pregnancy. Those women who reported that they wanted to wait two or more years before the next pregnancy were coded as wanting to delay a future pregnancy (category 2). Finally, women who reported that they did not want (any)more pregnancies were coded as no more (category 3). Women who reported wanting to wait until after marriage were excluded as we focused our analysis on women in union. We use the desire for a pregnancy in the future at baseline for the midterm pregnancy outcome and likewise we examine the midterm desire for a pregnancy in the analysis of the endline pregnancy outcome.

At each time period, all women were asked if they (or their partner) were currently using modern contraception to avoid a pregnancy. If they reported using a method, they were asked what method they were using. Modern contraceptive use was coded one if the woman reported using long-acting methods (implant, IUD), short-acting or spacing methods (pills, condoms, injectables), or another modern method (lactational amenorrhea method (LAM) or spermicide).

### Statistical methods

We exploit the availability of two transition horizons (baseline-midterm and midterm-endline) that allow us to apply longitudinal regression methods to predict a subsequent birth by fertility intentions and modern contraceptive use at an earlier period. Specifically, we use a type of fixed effects estimation method that controls for possible correlation between explanatory variables such as the fertility intentions variables and unobserved time invariant characteristics of the respondents. Methods that do not control for this possible correlation will result in biased parameter estimates. The method we use is referred to as a correlated random effects model [13–15]. The use of correlated random effects allows us to directly test to see if its use is required through the application of a robust version of the Hausman test for endogenous regressors (see, for example, [16], page 132).

Using a longitudinal regression strategy allows us to develop a detailed understanding of the interrelationships of these variables as well as their ultimate impact on contraceptive use and pregnancy. Models were restricted to women who were in union. Additional covariates include age, education, religion, and parity. An interaction term between fertility intentions and modern contraceptive use was considered in a separate model. The classifications of the control variables and their distributions at baseline, midterm, and endline are shown in Table 1. This paper extends an earlier paper [10] that simply examined the transition from baseline to midterm. This paper includes the additional transition horizon (midterm-endline) which allows for more robust modeling.

Further analyses estimate the causal effect of fertility intentions and contraceptive use on reported intendedness of a subsequent birth or pregnancy. Using a correlated random effects multinomial logit regression model, we estimate a model for whether a woman had an intended pregnancy, an unintended pregnancy, or no pregnancy based on her fertility intentions and modern contraceptive use at an earlier period. Covariates also include age, education, religion, and parity. A separate model includes the interaction term between fertility intentions and modern contraceptive use. All models were performed using the Stata Statistical Software (Version 15). The binary outcome was run using the *xtlogit* command and the polytomous outcome was run using the Stata add on *GLLAMM*. All study procedures, consent materials, and data collection tools were reviewed and approved by the Institutional Review Board at the University of North Carolina at Chapel Hill and the Comité National d’Ethique pour la Recherche en Santé in Senegal.

## Results

### Descriptive characteristics of sample

At baseline, over 87% of women eligible for interview were surveyed in the three study cities; in the longitudinal sample, 75% of eligible baseline women from Pikine were surveyed at endline and the corresponding percentages for Guédiawaye and Mbao are 73 and 68%, respectively. We first present weighted percentages of demographic characteristics, contraceptive use, and subsequent birth or pregnancy for the full longitudinal sample of women in union surveyed at baseline, midterm, and endline in the three urban sites, as well as characteristics for the balanced longitudinal sample (Table 1). As expected, by endline, the longitudinal sample has aged such that a small percentage is in the youngest age group and a greater percentage is in the oldest age group (true in both the full sample and the longitudinal analysis sample). Correspondingly, a greater percentage of women have any children at midterm and endline than at baseline, although the difference is small in these variables for the two-year follow-up periods. Modern contraceptive use increased over time, with the largest increase in long-acting methods from about 3% at baseline to about 15% at endline. However most modern contraceptive users reported using short-acting methods including pills, condoms, or injectables at each survey period.

Table 1 also presents the future fertility intentions at each survey period. Focusing on the balanced longitudinal sample, we see that at baseline, 42% of women want to get pregnant in the next two years, 37% want to delay their next birth, and 21% do not want any more children. By midterm, the desire for no more children is 26.7% and by endline, this rises to 30%. Overall, about half of women had a subsequent birth or pregnancy between baseline and midterm. Further, between midterm and endline more than two-fifths of women had a birth/pregnancy.

### Bivariate associations

Table 2 presents fertility intentions and contraceptive use stratified by subsequent birth/pregnancy outcomes for the two survey transition periods (baseline-midterm, midterm-endline). We see some change in fertility intentions following a birth/pregnancy. Among women who reported an intended pregnancy since baseline, 41% had previously reported wanting to delay more than 2 years, and 6% initially reported wanting no more children. The majority of women who had an unintended pregnancy since baseline reported wanting to delay more than 2 years at baseline (61%), while 18% reported wanting no more children, and another 21% reported wanting a child now or within 2 years. Eighty percent of women who reported an unintended pregnancy were not using a modern method at baseline. Similar patterns are seen when comparing midterm intentions and contraceptive use by birth outcomes at endline. Among women who reported a pregnancy at endline as intended, 47% reported that they wanted to delay a pregnancy more than 2 years at midterm, and 8% of women with an intended pregnancy reported at midterm that they did not want any(more) children. Among women who had an unintended pregnancy, 21% reported wanting a pregnancy now at midterm. Use of short-acting or spacing methods at midterm was similar across women who had an intended, unintended, or no pregnancy by endline, but use of long-acting methods was highest (14%) among women who did not have a pregnancy.

**Table 2:** Comparison of fertility intentions and contraceptive use in prior period with pregnancy experience in 2-year follow-up period among women in union, stratified by time period

	Midterm – 2013 - outcome			Endline – 2015 - outcome		
	Intended	Unintended	No pregnancy	Intended	Unintended	No pregnancy
Sample size (N)	406	144	500	318	115	617
Fertility intentions for future pregnancy captured in prior period (baseline – 2011; or midterm − 2013)						
Want Now/≤2 years	52.30	21.04	39.19	44.34	20.71	37.02
Want to delay >2 years	41.27	61.33	26.89	47.48	59.27	25.50
No more	6.43	17.63	33.91	8.18	20.02	37.48
Method used during prior period (baseline – 2011; or midterm −2013)						
Non-user	74.68	80.20	58.92	67.04	67.63	53.81
Long-acting method	0.96	0.25	4.96	3.59	0.97	14.40
Short-acting method	22.02	16.69	31.01	25.02	28.06	29.05
Other modern	0.21	0.83	0.04	0.95	1.49	0.0
Traditional method	2.14	2.03	5.07	3.40	1.85	2.73

Notes: use midterm and endline weights to weight by outcome period

### Multivariate results

For the binary outcome of birth/pregnancy experience in the two-year follow-up period, we used a correlated random effects logit model and for the polytomous outcome of pregnancy experience and intentionality of pregnancy we used correlated random effects multinomial logit. For each model, we estimated a version with and without interaction terms. Thus, we report coefficient estimates for a total of four models. In each case, we performed the robust version of the Hausman test of the null hypothesis that all explanatory variables were exogenous. This null hypothesis was strongly rejected for all four models with very small *p*-values (*p* < 0.001). Results for the multivariate correlated random effects logit for the binary outcome suggest that fertility intentions and contraceptive use in the prior period influence subsequent birth and pregnancy outcomes (Table 3). We demonstrate that women who want to delay a birth two or more years are less likely to get pregnant or have a birth in the follow-up period than women who want a pregnancy within 2 years (β:-1.43; 95% CI: − 1.93, − 0.94). Further, women who want no more children are also less likely to get pregnant or have a birth than women who want a pregnancy soon (β:-2.66; 95% CI: − 3.59, − 1.73). Women using modern contraception at the prior period are also less likely to get pregnant over the follow-up period than non-users (β:-2.12; 95% CI: − 2.64, − 1.59).

**Table 3:** Correlated random effect logistic regression models measuring subsequent birth/pregnancy experience by previous fertility intentions and contraceptive use among women in union at baseline, midterm, and endline

	Model 1			Model 2 (interactions)		
	Coefficient	Robust SE	95% CI	Coefficient	Robust SE	95% CI
Fertility intentions						
Wants now/≤ 2 years	Ref	Ref	Ref	Ref	Ref	Ref
Wants in + 2 years	-1.43***	0.25	−1.93, −0.94	−1.02***	0.31	−1.62, − 0.43
Does not want	−2.66***	0.47	−3.59, − 1.73	− 1.81***	0.54	−2.88, − 0.75
Modern contraceptive use						
No modern method	Ref	Ref	Ref	Ref	Ref	Ref
Modern method use	−2.12***	0.27	−2.64, −1.59	−1.27***	0.40	− 2.05, − 0.49
Interaction terms						
Use*Wants now/≤ 2 years				Ref	Ref	Ref
Use* Wants in + 2 years				−0.89 ^β^	0.47	−1.81, 0.03
Use*Does not want				−1.92**	0.66	−3.21, 0.63
Age group						
15–19 years	Ref	Ref	Ref	Ref	Ref	Ref
20–24 years	1.23	0.89	−0.51, 2.93	1.22	0.89	−0.53, 2.97
25–29 years	1.58	1.00	−0.37, 3.53	1.54	1.00	−0.41, 3.50
30–34 years	2.31*	1.07	0.20, 4.42	2.28*	1.08	0.17, 4.39
35–39 years	2.19 ^β^	1.14	−0.05, 4.43	2.19 ^β^	1.15	−0.06, 4.43
40+ years	2.33 ^β^	1.32	−0.26, 4.93	2.27 ^β^	1.33	−0.33, 4.88
Parity						
0	Ref	Ref	Ref	Ref	Ref	Ref
1	−1.65**	0.63	−2.89, − 0.41	− 1.86**	0.64	−3.12, − 0.60
2	−4.16***	0.86	−5.85, − 2.48	−4.42***	0.87	−6.12, − 2.72
3	−6.67***	1.05	−8.73, − 4.61	−6.93***	1.06	−9.01, − 4.85
4	−9.26***	1.25	− 11.72, − 6.81	−9.56***	1.25	− 12.03, −7.08
5+	− 10.56***	1.40	− 13.29, −7.82	− 10.75***	1.41	− 13.51, − 8.00
Education						
None	Ref	Ref	Ref	Ref	Ref	Ref
Primary	−0.09	0.52	−1.10, 0.93	− 0.08	0.52	−1.09, 0.94
Secondary +	1.25	0.80	−0.33, 2.82	1.27	0.81	−0.32, 2.86
Muslim	−0.18	1.56	−3.23, 2.88	−0.24	1.56	−3.30, 2.81

^β^*p* < 0.10; *p < 0.05; **p < 0.01; ***p < 0.001

Sample size = 2098 observations; 1050 individuals

In the model including an interaction between contraceptive use and fertility intentions (Table 3, Model 2), we find significant interaction effects. As above, fertility intentions matter such that those who want to delay a pregnancy and those who do not want to get pregnant are less likely to get pregnant than those who want to have a child soon. That said, the interaction effect demonstrates that women who want to delay a pregnancy and are also using a modern method are less likely to get pregnant. Further, women who do not want any more children and are using a method are the least likely to have a pregnancy in the follow-up period. The control variables in the models demonstrate that those women who have more pregnancies are less likely to have a subsequent pregnancy in the follow-up period and older women (ages 30+) are more likely to have a pregnancy in the follow-up period than women in union ages 15–19 years. Education and religion were not significant in any of the models.

The correlated random effects multinomial logit model results (Table 4) show that women who wanted to delay a pregnancy and those who do not want any(more) children and women using modern contraception are less likely to get pregnant (intended or unintended); these results are similar to those presented in Model 1 of Table 3. The last column of Table 4 shows that women who wanted to delay a pregnancy two or more years were significantly more likely to report a pregnancy as unintended compared to women who wanted a pregnancy soon. Further, women using a modern method of contraception in the previous period are less likely to have an unintended pregnancy (*p* < 0.10). This is not surprising, given that modern contraceptive use was higher among women who reported an intended pregnancy at midterm, and modern method use was the same for women reporting an intended and unintended pregnancy at endline (Table 2).

**Table 4:** Correlated random effect multinomial logistic regression models measuring the effect of fertility intentions on subsequent pregnancy/birth intendedness among women in union

	Intended pregnancy vs. No pregnancy			Unintended pregnancy vs. No pregnancy			Unintended vs. Intended pregnancy		
	Coef.	SE	95% CI	Coef.	SE	95% CI	Coef.	SE	95% CI
Fertility intentions									
Wants now/≤ 2 years	Ref	Ref	Ref	Ref	Ref	Ref	Ref	Ref	Ref
Wants in + 2 years	−1.55***	0.26	−2.06, − 1.05	− 0.92**	0.35	− 1.61, − 0.24	0.63*	0.29	0.05, 1.20
Does not want	−2.83***	0.51	− 3.83, − 1.84	−2.20***	0.60	− 3.37, − 1.02	0.64	0.51	− 0.36, 1.64
Modern contraceptive use									
No modern method	Ref	Ref	Ref	Ref	Ref	Ref	Ref	Ref	Ref
Modern method use	−2.01***	0.28	− 2.56, −1.46	−2.48***	0.34	−3.15, − 1.81	−0.47^β^	0.27	− 1.01, 0.07

^β^*p* < 0.10; *p < 0.05; **p < 0.01; ***p < 0.001

Notes: Models control for age, parity, education, and religion

Table 5 demonstrates that results for the models that include an interaction between fertility intentions and modern contraceptive use were similar to results presented in Model 2 of Table 3. Specifically, women who wanted to delay or avoid a pregnancy and were using modern contraception were more likely to have no pregnancy than to experience an unintended pregnancy (negative and significant coefficients). Similarly, women who did not want to get pregnant and were using modern contraception were more likely to have no pregnancy than to experience an intended pregnancy. When comparing women who reported an unintended pregnancy to women who reported an intended pregnancy, the interaction term between fertility intentions and contraceptive use was not significant and only intention mattered in the comparison such that women who wanted to delay were more likely to report an experienced pregnancy as unintended compared to women who reported that they wanted a pregnancy “now”.

**Table 5:** Correlated random effect multinomial logistic regression model with interactions measuring the effect of fertility intentions on subsequent pregnancy/birth intendedness among women in union

	Intended pregnancy vs. No pregnancy			Unintended pregnancy vs. No pregnancy			Unintended vs. Intended pregnancy		
	Coef.	SE	95% CI	Coef.	SE	95% CI	Coef.	SE	95% CI
Fertility intentions									
Wants now/≤ 2 years	Ref	Ref	Ref	Ref	Ref	Ref	Ref	Ref	Ref
Wants in + 2 years	−1.18***	0.31	− 1.79, −0.57	−0.44	0.43	− 1.28, 0.40	0.74*	0.36	0.04, 1.44
Does not want	−1.94***	0.58	−3.09, −0.80	−1.45*	0.69	−2.80, − 0.10	0.50	0.59	−0.67, 1.66
Modern contraceptive use									
No modern method	Ref	Ref	Ref	Ref	Ref	Ref	Ref	Ref	Ref
Modern method use	−1.25**	0.40	−2.04, −0.46	− 1.42*	0.61	− 2.61, − 0.22	− 0.17	0.53	−1.21, 0.88
Interaction terms									
Use*Wants now/≤2 years	Ref	Ref	Ref	Ref	Ref	Ref	Ref	Ref	Ref
Use* Wants in + 2 years	−0.79	0.48	−1.74, 0.15	− 1.19^β^	0.68	−2.52, 0.14	− 0.39	0.59	− 1.54, 0.75
Use*Does not want	−2.01**	0.73	−3.43, −0.59	−1.81*	0.87	− 3.51, − 0.10	0.20	0.81	− 1.38, 1.78

^β^*p* < 0.10; *p < 0.05; **p < 0.01; ***p < 0.001

Notes: Models control for age, parity, education, and religion

## Discussion

Our findings show that fertility intentions are fluid and that women who report that they do not want to get pregnant or want to delay a pregnancy may report the intentionality of an experienced pregnancy as intended post hoc; this may also happen in the opposite direction – women go from intending a pregnancy to reporting an experienced pregnancy as unintended. This means that the common metric of gaps in family planning use – unmet need for family planning – may be misclassifying some women in need.

The correlated random effects analyses presented here take advantage of the longitudinal data to use an extension of fixed effects methods to control for the endogeneity of intentions and contraceptive use as predictors of birth outcomes. This has not been done in previous studies and so the results of previous research may be biased [10, 17, 18]. Using three time periods and controlling for this endogeneity we show that both fertility intentions and contraceptive use are related to subsequent pregnancy or birth experience. Women who are using contraception in the previous time period are the most likely to not get pregnant in the follow-up period. Further, women who want to delay or avoid childbearing are also less likely to get pregnant, although the distinctions between the desire to delay or avoid does not seem to differentiate these women in their pregnancy/birth experience. Finally a combination of fertility desires and contraceptive use is associated with pregnancy experience such that those women who want to delay or limit childbearing and are using modern contraception are the least likely to get pregnant followed by those who either want to delay or limit childbearing but aren’t using.

These results extend earlier analyses that used the Senegal baseline and midterm data [10] and improve on the methods in that paper by controlling for endogeneity in fertility intentions over time. The earlier analysis demonstrated that women who wanted to delay childbearing two or more years were the most likely to get pregnant (i.e., more likely than those women who wanted a birth soon or within 2 years) and modern method users were less likely to get pregnant than non-user [10]. Further, interactions demonstrated that desires to delay or avoid a pregnancy were only meaningful among women who also used contraception suggesting the importance of acting on fertility desires through modern method use. Similarly, in a synthesis of longitudinal studies on fertility preferences and subsequent childbearing in Africa and Asia, Cleland and colleagues (2019) demonstrate that the desire to stop childbearing is a powerful predictor of subsequent fertility whereas the desire to delay was not consistently related to subsequent fertility behaviors [19]. Conversely, in this analysis, we find that both contraceptive use and fertility intentions matter such that women who are using are less likely to get pregnant as are women who want to delay or limit childbearing. However, women who want to delay or limit and are using contraception are the most likely to avoid a pregnancy in the follow-up period. We see similar results when we compare women who had no pregnancy to women with intended or unintended pregnancies. Women who wanted to delay or limit childbearing and were also using a modern contraceptive method were the most likely to avoid pregnancy compared to both women with intended and unintended pregnancies.

This analysis also extends the earlier analysis by showing that there is little difference in fertility desires and contraceptive use between women who report a pregnancy experienced as intended versus unintended. We show that those women who report that they wanted to delay a pregnancy were more likely to report a pregnancy as unintended than intended compared to those who reported that they wanted to get pregnant soon. This may reflect post-hoc rationalization of intentionality of a pregnancy among women in this setting with high overall fertility (total fertility rate in 2015 was 4.9 – [6]). This finding is consistent with the recent synthesis of longitudinal studies that demonstrated that in settings with overall higher levels of contraceptive use the consistency between preferences and subsequent reproduction increases [19]; this may reflect a better ability to act on intentions in settings with more use and stronger social norms supporting use.

This paper has strengths and limitations. Among the strengths is that we use data from three time periods which permits the use of lagged variables (fertility intentions and contraceptive use) in a correlated random effects model to control for endogeneity of some of the explanatory variables. This analysis is not without limitations. First, women may change their fertility intentions over time [19] and thus using the lagged fertility intention may not accurately reflect the actual fertility desire at the time of the pregnancy experienced. Second, the sample was limited only to women who had observations from the three time periods and thus those women who were not found may be different than the women surveyed at all three time points. With the data available, we demonstrate that the full baseline sample of women in union is similar to the reduced baseline sample, however, if the women who were not found differ longitudinally from the found sample, we are unable to control for this potential bias. Third, the data for this study come from three urban areas of Senegal. Thus, the results may not be generalizable to all urban areas in Senegal nor to other non-urban sites in Senegal.

## Conclusions

These analyses demonstrate that fertility intentions are meaningful: women who want to delay or avoid childbearing are less likely to get pregnant. Those women who act on fertility intentions by using modern contraception are the least likely to get pregnant. Women who do not act on their intentions put themselves at risk of a pregnancy. These women may be more ambivalent toward a future pregnancy or toward contraceptive use. Future research using qualitative methods could be useful among women who report that they do not want to get pregnant and are not using contraception (e.g., have an unmet need for contraception). Information from these women may help to identify barriers to use and potentially offer solutions to address identified barriers whether they relate to social norms around use; problems with the provision of services; or the need for design of novel contraceptive methods that better meet women’s and couple’s needs. Program planners and policy makers seeking to develop future family planning programs in Senegal and elsewhere in sub-Saharan Africa should focus on encouraging women to act on their fertility intentions, especially in contexts like Senegal with high fertility. As women begin to desire fewer children and recognize their ability to act on this desire, it is expected that family planning use will rise with a concomitant decline in fertility and unintended pregnancies. This should lead to improved health and well-being for all women, children, and families.

## Data Availability

Restricted data are available for download after an approval of a restricted use application, which involves signing a data use agreement and providing brief information about intent of use. Information can be found at: 10.15139/S3/12KUC0.

## Abbreviations

DHS: Demographic and Health Survey
LAM: Lactational amenorrhea method
MLE: Measurement, Learning & Evaluation
URHI: Urban Reproductive Health Initiative
PSUs: Primary sampling units
